# Some Spinal Cord Lesions

**Published:** 1911-04-22

**Authors:** 


### NEUROLOGY.

### SOME SPINAL CORD LESIONS.

### II.—AMYOTROPHIC LATERAL SCLEROSIS.

In this disease weakness and spastic rigidity of the legs are associated with atrophy and weakness of the muscles of the hands and forearms. It is due to the combined lesions of progressive muscular atrophy and primary spastic paraplegia—*i.e.* a degeneration of the anterior cornua in the lower cervical region and sclerosis of the pyramidal tracts throughout the spinal cord and medulla. It rarely affects persons under twenty-five years of age, and according to Charcot is commoner in men than in women.

The onset is gradual, the earliest symptoms being weakness and wasting of the muscles of the hands. When the disease is well marked the patient may come under observation for weakness and rigidity of the legs, or for atrophy of the muscles of the hands, or for both conditions. The chief signs of the disease are weakness of the muscles of the hands and arms, and, after a time, contracture of the wasted muscles. The legs also become weak and rigid. Fibrillary contractions of the atrophied muscles are common. There are no sensory disturbances of any kind.

At first both wrist and elbow jerks may be increased, but as the atrophy progresses and fresh muscles are involved they become diminished, and later on cannot be obtained. The knee-jerks are exaggerated, and ankle clonus and extensor plantar reflexes can be elicited. Wasting begins in the muscles of the hands, but soon spreads to the arms. Later in the course of the disease atrophy of the muscles of the legs may occur, but it is by no means a constant change. Sometimes tongue wasting ensues if the degeneration ascends to the medulla and produces bulbar paralysis.

Excitability to both the interrupted and continuous currents is diminished, and when the atrophy is well marked reaction of degeneration may be present in the affected parts of the hands and arms. The vesical and rectal functions are not affected. The degenerative changes in the anterior cornua may gradually extend upwards until the tongue, lips, and palate become paralysed and atrophied so that speech and deglutition are affected. Death may occur from bulbar paralysis.

The presence of the muscular atrophy distinguishes this disease from primary spastic paraplegia. Whenever investigating a case of spastic paraplegia see whether there is atrophy of the muscles of the hands, and whenever investigating a case of atrophy of the muscles of the hands examine the legs for weakness, rigidity, and exaggerated reflexes. It is distinguished from cervical myelitis by the slow, indefinite onset and the absence of sensory symptoms and of bladder and rectum disturbances.

